# Predictive and prognostic impact of tumour-infiltrating lymphocytes in triple-negative breast cancer treated with neoadjuvant chemotherapy

**DOI:** 10.3332/ecancer.2017.759

**Published:** 2017-08-15

**Authors:** Carmen Herrero-Vicent, Angel Guerrero, Joaquin Gavilá, Francisco Gozalbo, Abraham Hernández, Sergio Sandiego, Maria Asunción Algarra, Ana Calatrava, Vicente Guillem-Porta, Amparo Ruiz-Simón

**Affiliations:** 1 Medical Oncology Department, Valencian Institute of Oncology, 46008 Valencia, Spain; 2 Pathology Department, Valencian Institute of Oncology, 46008 Valencia, Spain

**Keywords:** triple-negative breast cancer, tumour-infiltrating lymphocytes, predictive, prognostic value

## Abstract

**Introduction:**

In locally and locally advanced triple-negative breast cancer (TNBC), neoadjuvant chemotherapy (NAC) only induces a pCR in 30–35% of patients. Clinical and pathological factors are not enough to distinguish the patients who have no chance of a pCR or not. The tumour microenvironment is critical for cancer and tumour-infiltrating lymphocytes (TIL). Moreover, the NAC scenario is the perfect setting to study possible changes in TIL levels.

**Material and methods:**

Using our prospective maintained breast cancer (BC) database, we identified 164 TNBC patients treated with NAC between 1998 and 2015 with enough samples of diagnostic biopsy and after surgery. Evaluation of TILs before and after NAC followed a standardised methodology for visual assessment on haematoxylin–eosin sections and the amounts of TILs were quantitated in deciles. We categorised lymphocyte-predominant breast cancer cutoff according to a receiver operating characteristic (ROC) analysis. We categorised LPBC as involving > 40% lymphocytic infiltration tumour stroma. The primary end point was predictive value of TILs to NAC, and the secondary end point was disease-free survival (DFS). DFS was analysed using the Kaplan–Meier method and the groups were compared with a long-rank test. Univariate and multivariate Cox models were used to generate hazard ratios for determining associations between variables such as TIL after NAC and DFS.

**Results:**

A total of 164 TNBC patients were treated with NAC and surgery. The main patients’ characteristics are listed in Table 1. We identify different pathological complete response to anthracycline and taxane-based NAC; LPBC subgroup 51 from 58 patients (88%) pCR versus non- lymphocyte-predominant breast cancer (LPBC) subgroup 10 from 106 (9%) pCR, *p* = 0.001. At a median follow-up of 78 months, LPBC was associated with better DFS; the three-year Kaplan–Meier estimates for DFS were 2% and 30 % for patients with LPBC and non-LPBC, respectively, *p* = 0.01. Univariate and multivariate analysis confirmed TIL to be an independent prognostic marker of DFS.

**Conclusions:**

Tumour-infiltrating lymphocytes could be routinely used in locally advanced TNBC treated with anthracycline and taxane, such as biomarker, to be enabled the identification of different two subgroups: LPBC patients have a very high response to NAC pCR 88%, meanwhile non-LPBC patients only achieve 9%. Moreover, non-LPBC patients have a worse prognosis than LPBC patients. This data verified the predictive and prognostic value of TIL.

## Introduction

Triple-negative breast cancer (TNBC) is a subtype of breast cancer with a really bad prognosis. In locally advanced TNBC treated with neoadjuvant chemotherapy (NAC) only 30–35% of patients with TNBC get pCR, defined as no residual invasive carcinoma in breast (ypT0 or ypTis) and negative lymph node status (ypN0) [1]. Nowadays, NAC therapy is based on the patient´s characteristics, tumour size, nodes, oestrogen and progesterone receptors, overexpression of human epithelial growth factor receptor, grade and ki67 [2]. However, these factors are not enough to distinguish the patients who have no chance of a pCR or not [1].

Recent studies suggest that the tumour microenvironment is critical for survival, invasive growth, and metastasis of the cancer [3]. Indeed, the host immune system responds to fight cancer; normal breast tissue does not contain aggregates of immune cells, but breast tumours and stroma display higher levels of immune cells infiltrates [4]. The median percentage of stromal tissue infiltrated with tumour-infiltrating lymphocytes (TIL) in TNBC was 20% in TNBC [5].

TNBC treated with NAC is the perfect setting to study possible changes in TIL levels, since we have a histological sample pre-treatment after NAC. Basal TILs or pre-NAC could act as such a predictive marker for response to standard NAC [6–8]. Furthermore, in patients without pCR, TILs in residual carcinoma could help in assessing risk. After all, new strategies should be considered to improve the outcome of TNBC patients [1, 9].

Criteria to evaluate TILs are different depending on the study. Cut-offs to divide between lymphocytic-predominant breast cancer (stromal TILs > 50%) were used in some studies [6–8], while others used different cut-offs [5, 8, 10]. In 2015, an international TIL breast cancer working group recommended a scoring system for TILs involving haematoxylin–eosin (HE) staining for routine practice [11]. We aimed to investigate the utility of TILs using this scoring approach.

## Materials and methods

### Patients

A retrospective, single-institution study was conducted at the Valencian Institute of Oncology with ethics committee approval. The study is based on a database maintained for patients with locally advanced breast cancer who received neoadjuvant treatment between 1998 and 2015.

Patients were included in the study if they had TNBC [oestrogen receptor-, progesterone receptor-, and human epidermal growth factor receptor type 2- (ER- PR- and HER2-)] The threshold for ER and PR positivity was set at 1% using immunochemical staining. HER2 was positive if tumour cells showed 3+ by IHC or 2+ by IHC with amplification using silver *in situ* hybridisation. Patients were treated with neoadjuvant chemotherapy (NAC) based on anthracycline and taxane regimen.

Clinicopathological information was obtained from the database: age, histological classification, grade, tumour size, lymph node status, ki67, lymphovascular invasion, response to NAC and adjuvant treatment.

### Samples

Core biopsies before NAC and surgical specimens obtained after NAC were reviewed. The largest size of tumours, histologic type and grade, lymphovascular invasion, proportion of ductal carcinoma in situ (DCIS), number of positive lymph nodes, and treatment response in breast and lymph nodes were evaluated. Tumour size and extent in breast and lymph nodes were assessed according to the recommendation proposed by Provenzano and the histologic type and grade were defined in accordance with the World Health Organisation classification and classified using the modified Scarff–Bloom–Richardson grading system, respectively.

HE samples were reviewed by a breast pathologist (FG) who was blinded to the patient profiles. He defined TIL score as the proportion of the stromal area infiltrated by lymphocytes following the recommendations of the International TIL Breast Cancer Working Group [7].

Ninety-six per cent of stored HE samples were retrieved for TIL assessment. A representative slide containing a relatively high amount of lymphocytic infiltration around invasive cancer was selected for each patient. The evaluation of TILs was following a standardised methodology for visual assessment on HE sections and the amounts of TILs were quantitated in deciles. Due to the heterogeneity of TILs, with different intensities of lymphocytes in different areas, hot spots at the invasive edge were avoided.

We define ‘cTIL’ as TIL in core biopsies before NAC and from patients without pathological complete response ‘ypTIL’ as TIL in surgical specimens obtained after NAC.

### Outcomes

Predictive information was obtained from electronic charts. Pathological complete response (pCR) was defined as no residual invasive carcinoma in breast (ypT0 or ypTis) and negative lymph node status (ypN0).

Prognostic information was retrospectively obtained from a maintained clinical database. Disease free survival (DFS) was defined as the period of time between surgery and breast cancer relapsed, death of any cause or latest follow-up.

### Statistical analysis

Data were analysed using SPSS version 20.0.

We categorised lymphocyte-predominant breast cancer (LPBC) cut-off according to a COR analysis.

The association between clinical and pathological parameters was tested with χ^2^ test for categorical variables. Mean differences were studied with the *t*-test.

To identify variables associated with an increased recurrence, we performed firstly Kaplan–Meier curves. All events were measured from the date of histological diagnosis. The statistical significance between survival curves was determined by a log-rank test between two groups. Then, significant variables were included in a multivariate analysis using Cox proportional hazards. The median follow-up period for surviving patients was 78 months. All tests were two tailed, and *p* < 0.05 was significant.

## Results

### Patients´ characteristics

A total of 756 patients diagnosed with locally advanced invasive carcinomas and treated with NAC followed by surgery were identified from 1998 to 2015.

Based on the criteria described, 181 patients were diagnosed with TNBC, and of these, 164 patients (90.6%) had enough samples available before and after NAC.

The median age was 49 years (range 29–81). The main clinical and pathological patients’ characteristics are described in Table 1.

Fifty-nine per cent were treated with adriamicyn 60 mg/m^2^ – ciclofosfamide 600 mg/m^2^/21 days × 4 cycles followed by weekly taxane × 12 cycles. Twenty per cent of patients received three-weekly docetaxel. Three per cent of patients were not treated with anthracycline and 5% with taxane-based NAC. Fifty per cent were treated with breast conservatory surgery (BCS), but 5.4% re-resections were needed to get 99% negative margins; 37.2% of patients presented pCR (ypT0/ypTis ypN0); 88% received adjuvant radiotherapy. The NAC regimen and surgery approach are also detailed in Table 1.

### TILs analysis

The detailed distribution of basal TILs (before NAC) is detailed in Table 2. The main samples (57%) presented basal TILs from 1% to 10%.

The lymphocyte-predominant breast cancer (LPBC) cut-off was 40% (*p* = 0.001). We categorised LPBC as involving > 40% lymphocytic infiltration tumour stroma.

Fifty-eight patients (35.4%) were LPBC, meanwhile 106 patients (64.6%) were non-LPBC.

The detailed distributions of clinical and pathological characteristics are summarised in Table 2. There were no differences between median age, histology, clinical stage, lymphovascular invasion, ki67 marker, neither NAC regimen. However, LPBC group presented higher histological grade 3 (64% vs. 34% p = 0.005), higher BCS (67 % vs. 40%, *p* = 0.001) than non-LPBC.

#### Predictive value of TIL

Fifty-one of 58 (87%) patients from LPBC group and 10 from 106 (9.4%) from non-LPBC group achieved a pCR (Table 2). LPBC group presented higher pCR than non-LPBC (87 % vs. 9% *p* = 0.001). Breast tumour pCR (ypT0/is) and lymph nodes pCR (ypN0) were also higher in LPBC tan non-LPBC (86% vs. 12.2% *p* = .001) and (93% vs. 45%* p* = 0.001), respectively.

### Prognostic value of TIL

At a median follow-up of 78 months, 41 recurrences (25%) were observed after standard NAC and curative surgery; 36 recurrences (33.9%) in the non-LPBC group and five recurrences (8.5%) in LPBC (*p* = 0.005).

**Figure 1.**
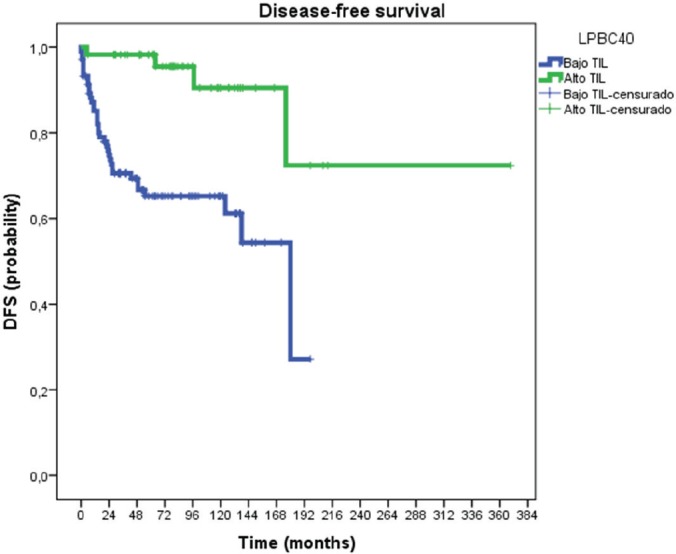
Disease-free survival curves according to LPBC and non-LPBC groups.

Figure 1 illustrates DFS curves according to LPBC and non-LPBC groups. TILs (high and low levels) proved to have significant prognostic value (*p* = 0.005) regarding DFS. Median DFS in non-LPBC was 20 months (IC 95% 10–29 months) and LPBC was 97 months (IC 95% 27–166 months). At 3 years, LPBC presented 2% of recurrences meanwhile non-LPBC presented 30% (*p* = 0.01).

By univariate analysis, the variables associated with an increased recurrence were clinical stage (cTNM), pCR (ypT0/is ypN0), TILs levels before NAC (cTNM), and after NAC-TILs levels (ypTILs).

By multivariate analysis, the variables associated with an increased recurrence were RCP (HR 10, *p* = 0.014) y ypTIL (HR 0.8, *p* = 0.016)

Univariate and multivariate analysis details are described in Tables 3 and 4.

## Discussion

In summary, we investigate the utility of TILs following the scoring system of an international TIL breast cancer working group [12]. In this retrospective study, we report the predictive and prognostic value of TILs in locally advanced TNBC treated with NAC based on anthracyclines and taxanes.

## Cut-off point

We recognised the heterogeneity of TILs cut-off in order to divide between LPBC and non-LPBC [5, 10, 11]. TILs were analysed as a continuous variable, and also as a dichotomous variable with a cut-off. Some studies defined LPBC (stromal TILs > 50%) [5, 8], while others used different cut-offs [10, 11]. For example, Hida *et al* [3] divided them into three different groups: the high-TIL (stromal and/or intratumoural TIL > 60%) intermediate-TIL (TIL from 10% to 60%) and low-TIL (TIL < 10%) groups were different. However, in Hida´s study, the difference between these three groups was not significant because of the absence of events in the high-TIL group.

In our study, we performed a ROC curve and we categorised LPBC as involving > 40% lymphocytic infiltration tumour stroma. However, the diversity and richness of the antitumour immune response is, of course, severely underestimated by assessment with H&E.9

## Predictive value

There are data which support that basal TIL or before NAC could be a predictive value in locally advanced TNBC treated with NAC based on anthracyclines [5, 9, 17].

Denkert *et al* [11] demonstrated this in a retrospective study of 1058 patients from GEPADUO and GEPARTRIO trials with breast cancer treated with neoadjuvant cancer with anthracyclines and taxanes. Patients with TIL levels higher than 60%, had higher rates of pCR compared to patients with low levels of TIL diagnosis (40% vs. 7.2%). Another study [3], in which 474 patients were included with locally advanced TNBC and Her2 treated with NAC, suggested that TNBC with high levels of TIL at diagnosis had higher pCR than patients with low levels of TIL (37% vs. 16%).

In the GEPARSIXTO study [15], patients with LPBC who were treated with carboplatin in addition to anthracycline and taxane had impressive pCR rates: 74% versus 43% compared with the pCR rate of patients with LPBC treated without carboplatin (*p* = 0.005).

**Figure 2.**
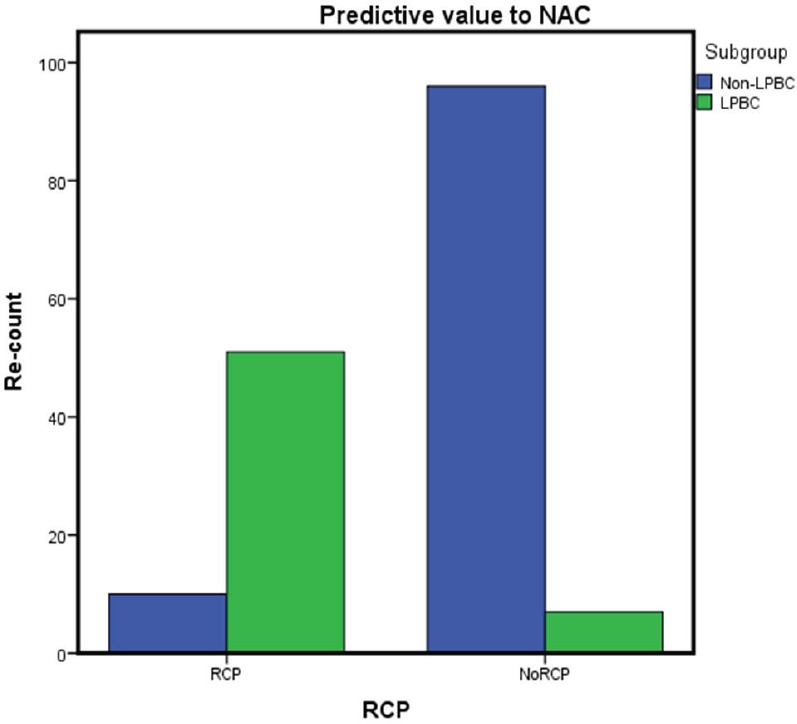
Pathological complete response to anthracycline and taxane-based NAC.

In our study, we identify different pCR to anthracycline and taxane-based NAC: LPBC subgroup 88% pCR versus non-LPBC subgroup 9% pCR, *p* = 0.001 (Figure 2).

TIL determination could be considered as a diagnostic in locally advanced TNBC patients treated with NAC based on anthracycline and taxanes because it represents a surrogate marker for efficacy [9].

Chemotherapy induces changes in the immune response, which are partially characterised, although some studies suggest that there could be cooperation between both of them [18–21]. In our study, the correlation between the two was evaluated, the targeted tumour belonging to the non-LPBC group did not change after receiving NAC.

## Prognostic value

TILs have been evaluated in the past 5 years in nearly 16.000 patients in prospective studies with clinical follow-up data available, which highlights a rapid accumulation of evidence [5, 7, 10, 17, 22]. The BIG-02-98 trial, with 2009 breast cancer patients and 256 with TNBC, suggested that higher TIL levels were a significant favourable prognostic factor because they were associated to higher DFS and OS [5].

Two different studies ECOG 2197 and 1199 with 481 TNBC patients confirmed that higher TIL levels were a favourable prognostic factor [7].

Dieci *et al* reported the prognostic value of TILs in residual disease after NAC in TNBC [8]. Their survival curves for the high-TIL (stromal and/or intratumoural TIL > 60%) intermediate-TIL (TIL from 10% to 60%) and low-TIL (TIL < 10%) groups were different. However, in Hida´s study, the difference between these three groups was not significant because of the absence of events in the high-TIL group [3].

Therefore, Simon *et al* [23] reported that the prognostic value of TILs in TNBC could be considered as Level-I evidence.

In our study, at a median follow-up of 78 months, LPBC was associated with better DFS; the three-year Kaplan–Meier estimates for DFS were 2% and 30 % for patients with LPBC and non-LPBC, respectively, *p* = 0.01. Moreover, univariate and multivariate analysis confirmed TIL to be an independent prognostic marker of DFS.

Although our findings may not change standard NAC options for locally advanced TNBC, TIL evaluation could be included in the standard histopathological practise as a prognostic factor.

## TILs in residual disease

Patients with residual disease following NAC in TNBC are considered a poor prognosis group [24]. The standard of care for patients with TNBC who have residual disease after NAC is observation. However, new effective therapies in reducing recurrences are unknown.

The combination of TIL after NAC and pCR could enable patient stratification in different groups. The presence of TILs in residual disease could stratify patients and confers a better prognosis in non-pCR with LPBC patients. However, tumour-intrinsic signalling pathways related to immunity remains poorly understood [25–27].

Patients with LPBC

- And pCR have an excellent outcome
- Patients who do not achieve pCR have a good outcome but might require further enhancement of immunity by targeting counter-regulatory mechanism that prevent tumour elimination.

Patients with non-LPBC

- And pCR also have a good outcome but might require therapies aimed at establishing immune infiltration by tumour-specific T cells, which could be achieved through creating a more permissive immune microenvironment.
- Patients who do not achieve pCR have worse outcomes and might benefit from more toxic approaches. In this subgroup, a combination for promoting a more functional immune microenviroment could be useful. Loi *et al* [26], showed a correlation between reduced infiltration of TILs in residual disease in TNBC following NAC and transcriptional activity in RAS/MAPK pathways. A synergistic combination therapy with PD/PDL-1 and MEK inhibitors in mouse models of TNBC has been described [26]. Two recent trials have evaluated the efficacy of antibodies directed at PD/PDL1 in patients with TNBC with promising results [28–30]. But biomarkers for immune-checkpoints inhibitors are not yet available.

## Conclusions

- Several recent clinical studies have evaluated TILs in TNBC patients with different methodological approaches. The aim of this study is to analyse the predictive value of TIL following the recommendations for TILs Evaluations in Breast Cancer by the International TILs Working Group.
- Clinicopathological biomarkers are not sufficiently accurate to distinguish the patients who have no chance of a pathologic complete response.
- In this retrospective study, we have identified different subgroups: LPBC with very high pCR response to NAC and non-LPBC with lacking pCR.

## Conflicts of interest

This research received a specific grant ‘Beca Mutual Médica 2016’ from Fundación Mutual Médica.

The authors declared they have no competing interests.

## Figures and Tables

**Table 1.**
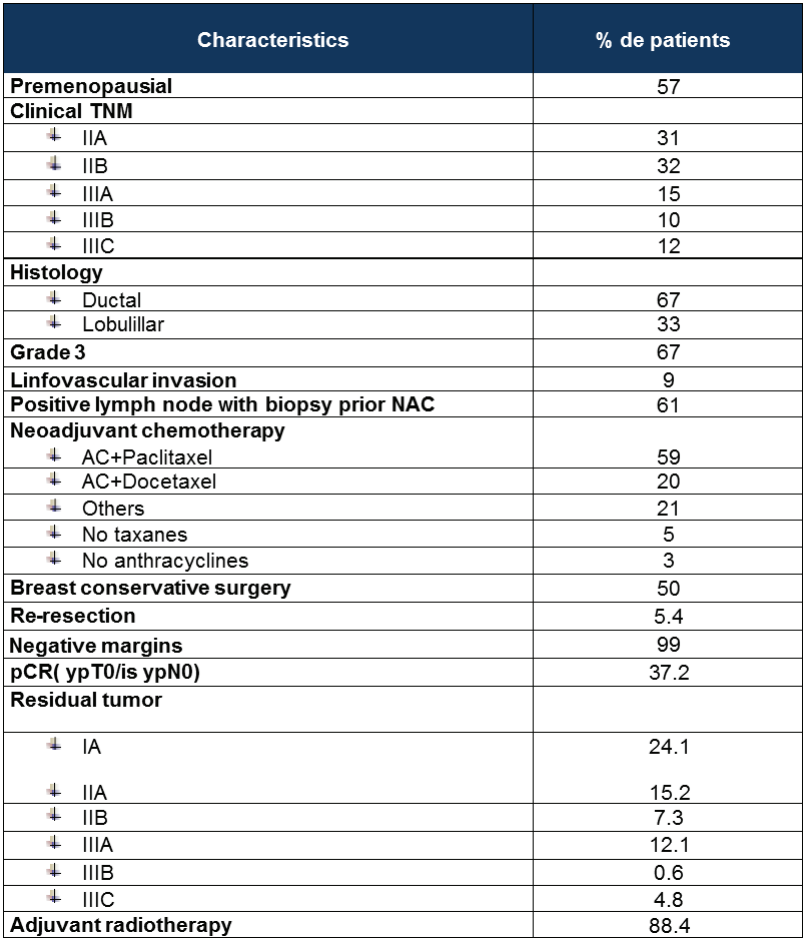
Baseline patient and tumour characteristics.

**Table 3.**
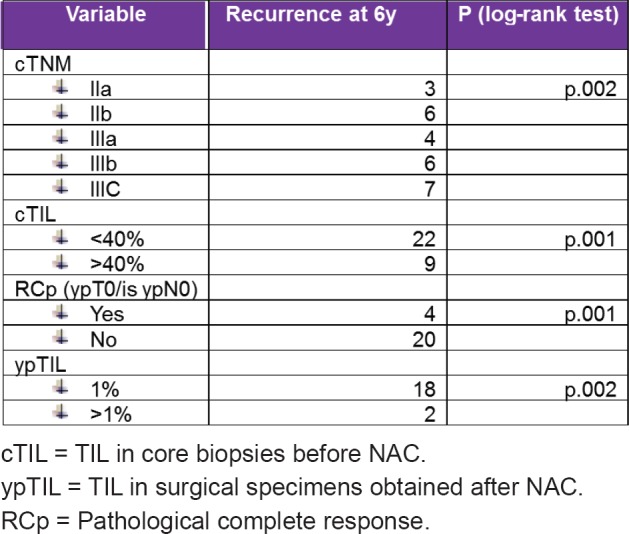
Univariate analysis details: variables associated with an increased recurrence.

**Table 4.**
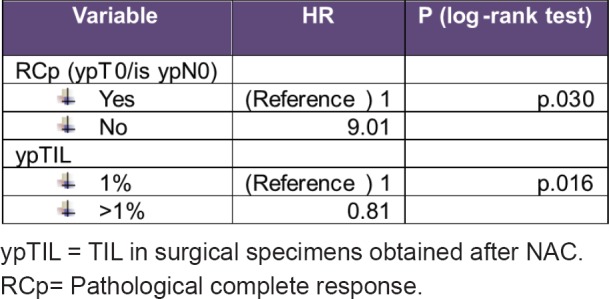
Multivariate analysis details: variables associated with an increased recurrence.

**Table 2.** Detailed distributions of clinical and pathological characteristics between LPBC group and non-LPBC group.

Variable	TILs level		χ^2^
	HIGH	LOW	
Age < 40 y	32 (55%)	60 (56%)	p.345
Stage TNM			
IIa	22 (38%)	29 (28%)	p.205
IIb	22 (38%)	32 (30%)	
IIIa	7 (12%)	18 (17%)	
IIIb	3 (5%)	10 (9%)	
IIIc	4 (7%)	17 (16%)	
Histology			
Ductal	33 (57%)	64 (60%)	p.060
Lobular	25 (43%)	42 (40%)	
Grade 3	37 (64%)	32 (30%)	p.005
DCIS	6 (10%)	7(6%)	p.486
LVI	6 (10%)	12 (11%)	p.106
Ki67>50	50 (86%)	90 (85%)	p.270
NAC			
Anthracycline+taxane	47 (81%)	98 (92%)	p.570
pCR (ypT0/is, ypN0)	51 (88%)	10 (9%)	p.001
ResidualTumor			
Multifocal	4 (7%)	24 (22%)	p.001
Conservative surgery	39 (67%)	43 (41%)	p.001
Adjuvant Radiotherapy	51 (88%)	93 (88%)	p.754
Quimioterapia adyuvante	12 (21%)	47(44%)	p.078
